# Neotropical *Thoasia* Liebke, 1939 and *Straneotia* Mateu, 1961 of the Cryptobatida group, subtribe Agrina: Taxonomic revisions with notes on their ways of life (Insecta, Coleoptera, Carabidae, Lebiini)

**DOI:** 10.3897/zookeys.742.22900

**Published:** 2018-03-12

**Authors:** Terry L. Erwin, Charlotte Aldebron

**Affiliations:** 1 Hyper-diversity Group, Department of Entomology, MRC-187, National Museum of Natural History, Smithsonian Institution, P.O. Box 37012, Washington D.C. 20013-7012, U.S.A.

**Keywords:** Brazil, Ecuador, French Guiana, Perú, Venezuela, rainforest trees, arboreality, biodiversity

## Abstract

*Thoasia* Liebke, 1939 and *Straneotia* Mateu, 1961 of the Cryptobatida group, subtribe Agrina, Lebiini, living in the Neotropics are diagnosed, described, and illustrated. Occurrences of known species ranges are limited to the northern and western Amazon Basin and Guyana Shield. The following species are described as new: *Thoasia surinamensis* Erwin & Aldebron, **sp. n.**, Suriname, *Thoasia pterosmaragdos* Aldebron & Erwin, **sp. n.**, French Guiana, *Thoasia manu* Erwin & Aldebron, **sp. n.**, Ecuador, Perú; *Straneotia cylindroceps* Erwin & Aldebron, **sp. n.**, French Guiana, *Straneotia confundis* Aldebron & Erwin, **sp. n.**, Ecuador, and *Straneotia moi* Aldebron & Erwin, **sp. n.**, French Guiana. Two of these species, *Thoasia manu* and *Straneotia moi* are known from adults collected by insecticidal fogging of lowland rainforest trees, and these trees are listed in their respective descriptions. The following species are redescribed: *Thoasia rugifrons* Liebke, 1939, French Guiana, Venezuela; *Straneotia freyi* Mateu, 1961, Brazil; *Straneotia amazonica* Mateu, 1961, Brazil.

## Abstract (es)

*Thoasia* Liebke, 1939 y *Straneotia* Mateu, 1961 del grupo Cryptobatida, de la subtribu Agrina, Lebiini, que viven en la región del Neotrópico, son diagnosticados, descritos, e ilustrados. Las distribuciones conocidas de las especies son limitadas a la cuenca amazónica y el Escudo guayanés. Las siguientes especies son descritas como nuevas: *Thoasia surinamensis* Erwin & Aldebron, **especie nueva**, Surinam; *Thoasia pterosmaragdos* Aldebron & Erwin, **especie nueva**, Guayana Francesa; *Thoasia manu* Erwin & Aldebron, **especie nueva**, Ecuador, Perú; *Straneotia cylindroceps* Erwin & Aldebron, **especie nueva**, Guayana Francesa; *Straneotia confundis* Aldebron & Erwin, **especie nueva**, Ecuador; y *Straneotia moi* Aldebron & Erwin, **especie nueva**, Guayana Francesa. Dos especies, *Thoasia manu* y *Straneotia moi*, se conocen por adultos colectados con la técnica de nebulización con insecticida de árboles de bosque lluvioso de tierra baja. Estos árboles son mencionados en las respectivas descripciones. Las siguientes especies son redescritas: *Thoasia rugifrons* Liebke 1939, Guayana Francesa, Venezuela; *Straneotia freyi* Mateu, 1961, Brasil; *Straneotia amazonica* Mateu, 1961, Brasil.

## Introduction

This treatment of two more genera in the Cryptobatida group, subtribe Agrina, continues our goal of revising all its known taxonomic elements (cf. *Valeriaaschero* – Erwin 2004; *Hybopteroides* – Erwin and Ball 2012; *Hyboptera* – Erwin and Henry 2017; *Aspasiola* – Kyana Pike, in prep; *Cryptobatis* – Charlotte Aldebron, in prep.). Mateu (1961) treated *Otoglossa* and *Pseudotoglossa*, but did not have recent canopy samples at the time, thus his work was incomplete from a present day viewpoint; however, his treatment does allow identification of species described up to that time. Those latter two genera and the remaining genera (*Onota* and *Cylindronotum*) that have never been revised and are very well represented in recent fogging samples will be treated in the near future. Our goal in these generic treatments is to make known recent findings from the field and the gathering together of historical literature with the hopes that museum and field-oriented systematists will extend our knowledge through discovery of additional specimens (and species representatives) in collections and in nature.

The species of the Cryptobatida group are highly varied in color and external-form attributes. Their way of life is also varied to the extent we know it. *Hybopteroides* adults prey on Embiids under the latter’s webbing, while *Hyboptera* adults do the same on Psocoptera under their webbing. Colorful *Cryptobatis*, *Otoglossa*, *Aspasiola*, and, likely, *Valeriaaschero* are found on bracket fungi on dead wood, or standing live trees. While *Onota*, *Cylindronotum*, and *Pseudotoglossa* are speciose and abundant in canopy fogging samples, other than their aboreality and seasonality, their way of life is unknown.

## Methods and materials

(modified from Erwin and Henry 2017)

As noted in several past contributions, methods and species concepts follow those previously described (Erwin and Kavanaugh 1981; Kavanaugh and Erwin 1991). The species validation and diagnosis format follows as closely as possible that suggested in Erwin and Johnson (2000). For measurements, images of the specimens were taken using a Leica M420 stereoscope coupled to an EntoVision system. The resulting image was processed using the software Cartograph version 7.2.5 by Microvision Instruments. The magnification on the zoom was set to calibrate the system and it is embedded into the file of the image. The image was then opened with the software program Archimed version 6.1.4, also by Microvision and the Measure tool was then used to determine the lengths and widths of the various parts. A total of 43 images were obtained. Measurements of length (ABL, SBL) and maximum width (MW) follow those of Ball (1972) and Kavanaugh (1979): ABL (apparent body length), measured from apex of labrum to apex of longer elytron (in adults of these genera, the abdomen sometimes protrudes beyond the elytral apex, thus the ABL often is larger than the SBL; SBL (standardized body length), equals the sum of the lengths of the head (measured from apex of clypeus to a point on midline at level of the posterior edge of compound eyes); PL (pronotum length) is measured from apical to basal margin along midline; EL (elytron length) is measured from apex of scutellum to apex of the longer elytron; and MW (maximum width) is measured across both elytra at their widest point with suture closed. HW (head width) is measured from extreme margin of protuberant eyes left to right. Note that not all specimens available were measured because more than 33 specimens were available, thus we limited “n” to 33 as a statistically valid sample size. Sexes were measured separately; we found slight differences among the species sexes, and hence we report measurements for both sexes in our Tables (see Appendix 1, 2). For an explanation of the measurements and their incorporation in Appendix 1, see Erwin (2011) and Erwin and Ball (2011). For the present study, we report the harmonic mean, as we believe it better reflects the central tendency than the arithmetic mean.

Attributes of the abdominal ventral sterna are referred to using the numbering system generally accepted in carabid studies, i.e., the sternum divided medially by the hind coxae is sternum II (the first being hidden) and the last visible is sternum VII (Liu et al. 2011). In a revision of the genus *Pericompsus* (Erwin 1974), a problem was encountered with the term “stria” for features of their punctate elytra (i.e., the so-called striae were *not* actually striae, rather they were rows of punctures). The result was the use of the term “interneur” to apply to the attribute lying between intervals. Through use of this term, one could describe the feature as interneur striate, punctate, striatopunctate, etc.

A similar problem exists for the proximal end of the median lobe of the male genitalia. In Snodgrass (1935), the term “phallobase” is used, and we have adopted it here (see Erwin and Zamorano 2014). Therefore, by extension, in Carabidae, we can say phallobase hooded (e.g., Lebiini, Pseudomorphini), phallobase of two parallel sclerotized struts (basal trechines and *Andinodontis*), phallobase of two uneven struts (*Bembidion*), etc. Kavanaugh (pers. comm.) points out that with struts there are still connecting membranes surrounding the struts forming a “bulb.” We have chosen the aedeagal illustration of a male *T. manu*, new species (Fig. 3C) to display the identifying code letters and these apply to all illustrations of male genitalia included herein.

This study includes 138 adult specimens of *Thoasia* and 6 adult specimens of *Straneotia*, all currently at the National Museum of Natural History, Washington, DC (NMNH). Among these specimens, one was received from the Florida Department of Agriculture (FSCA, Paul Skelley, Collection Manager). Primary type specimens of new species will be deposited in their countries of origin if required by legal agreements, or museums of ownership at the conclusion of our studies on the Cryptobatida group.

The enhanced habitus images of the adult beetles portray most of the character states referred to in the key provided. Illustrations of male genitalia are standard for descriptive taxonomy of carabid beetles in both preparation and aspects presented, as is the presentation of the female genitalia (one example per genus, in this case *T. manu*, new species and *S. cylindroceps*, new species). All images were made with a Visionary Digital high resolution imaging system rendered using Photoshop to become “Digital Photo-illustrations.” Figure captions include an ADP number, which is a unique identification number for the specimen that was imaged and links the specimen and associated illustrations and/or images to additional information, such as collecting notes, in electronic databases at the NMNH.

Geographical data are presented for species based on all known specimens available at the time of manuscript preparation, including those in the literature. Geo-referenced data have been determined from locality information provided on specimen labels; only those exact geo-references reported in decimal degrees that are provided on the label are placed in quotes. Otherwise, we have estimated others as closely as possible from places, mileage, or other locality data listed on the label and searched with Google Earth Pro. Latitude and longitude for those are reported in decimal degrees and have been corrected from those reported on the labels, if necessary; our bottom line is that georeferenced locality data reported herein are far more accurate than those provided on specimens labels.

A distribution map is provided for the species of *Thoasia* and *Straneotia* (Fig. 7). Here, vernacular names in English are proposed, as common names are becoming increasingly needed in conservation reports and studies, and/or agricultural and forestry applications. These names are based on criteria set forth in Erwin (2011a) and applied in Erwin (2011b).

Host occurrences of rainforest trees are reported using the names provided by botanists who inventoried two fogging transects established by the senior author (TLE) in Ecuador. These names have not been elaborated with author names herein, as is traditional in botanical literature, however, they can be readily found on the internet.

## Accounts of taxa

### Tribe Lebiini

#### Subtribe Agrina

##### Cryptobatida Group (Modified from Erwin and Henry 2017)

**Diagnosis.** Adults. Head ventrally without suborbital setigerous punctures, neck not markedly narrowed, except somewhat in *Thoasia*. Mandible widened near base, scrobe wide, lateral margin markedly rounded; dentition of occlusal margins reduced, typical for Lebiini (cf. Shpeley and Ball 2001: figs 6–9); palpi with ultimate articles sub-securiform or securiform, paraglossae broad, glabrous, adherent, extended to anterior angle of glossal sclerite. Elytron usually with transverse depression at anterior third, appearing deformed; penultimate setigerous puncture of elytron umbilicate series not displaced laterally, nor medially. Posterior tibial spurs subequal, their margins smooth; tarsomere 4 usually bilobed. With or without a flagellum on the male endophallus.

**Notes.** Comb-claws, a prominent feature of many canopy and understory carabids are of varied distribution in this Group.

###### Key to the genera of the Cryptobatida Group, Subtribe Agrina (Lebiini)

(Enhanced from Erwin 2004, Erwin and Ball 2012, and Erwin and Henry 2017)

**Table:** 

1	Elytron at basal third depressed, surface uneven, tuberculate or not, and/or margin of pronotum angulate or sub-angulate at mid-lateral setiferous pore, or tubular	**Cryptobatida Group...2**
1’	Elytron neither depressed nor tuberculate, surface smooth with normal interneurs and intervals (or interneurs effaced); side of pronotum evenly rounded	**Plochionida Group, Calleidida Group, Agrida Group**
2(1)	Elytron markedly tuberculate overall or with a series of small setiferous tubercles in intervals 3 and 5	**3**
2’	Elytron without trace of discal tubercles; lateral marginal intervals with or without calli	**6**
3(2)	Prothorax somewhat tubular, much narrower than head	***Otoglossa* Chaudoir, 1872**
3’	Prothorax wider than head	**4**
4(3’)	Sides of pronotum narrowly reflexed except at mid-lateral seta, there wide; neck well-defined, narrow; elytra metallic blue, fore-body all or mostly rufous	***Thoasia* Liebke, 1939**
4’	Sides of pronotum broad and margins broadly reflexed throughout; neck broad; color otherwise	**5**
5(4’)	Elytron with numerous tubercles in at least three intervals; head dorsum transversely rugose; side margins of pronotum subtly angulate, or not; labrum large, broadly bilobed; tarsomere 4 bilobed	***Hyboptera* Chaudoir, 1872**
5’	Elytron not tuberculate; head dorsum longitudinally strigose (wrinkled); side margins of pronotum markedly angulate; labrum normal, rectangulate, slightly emarginate or truncate apically; tarsomere 4 not bilobed	***Hybopteroides* Erwin & Ball, 2012**
6(2’)	Antennomere 4 glabrous except for apical ring setae	**7**
6’	Antennomere 4 multisetiferous from basal third to apex, in addition to apical ring setae	**8**
7(6)	Elytron laterally with callus at apical third; male endophallus without flagellum	***Valeriaaschero* Erwin, 2004**
7’	Elytron laterally without callus at apical third; male endophallus with flagellum	***Aspasiola* Chaudoir, 1877**
8 (6’)	Head markedly narrow, elongate, and tubular; eyes more or less flat	***Straneotia* Mateu, 1961**
8’	Head normal with large hemispheric eyes	**9**
9(8’)	Head and pronotum densely and evenly punctate	***Cylindronotum* Putzeys, 1845**
9’	Head and pronotum smooth	**10**
10(9’)	Pronotum with lateral margin narrowly reflexed from base to apex	***Pseudotoglossa* Mateu, 1961**
10’	Pronotum with lateral margin moderately or markedly reflexed from base to apex	**11**
11(10’)	Elytron laterally at apical third with large callus	***Cryptobatis* Eschscholtz, 1829**
11’	Elytron laterally at apical third without callus	***Onota* Chaudoir, 1872**

###### Revision of the genus *Thoasia* Liebke, 1939

####### Thoasia

Taxon classificationAnimaliaColeopteraCarabidae

Liebke, 1939

**Figure 1.**
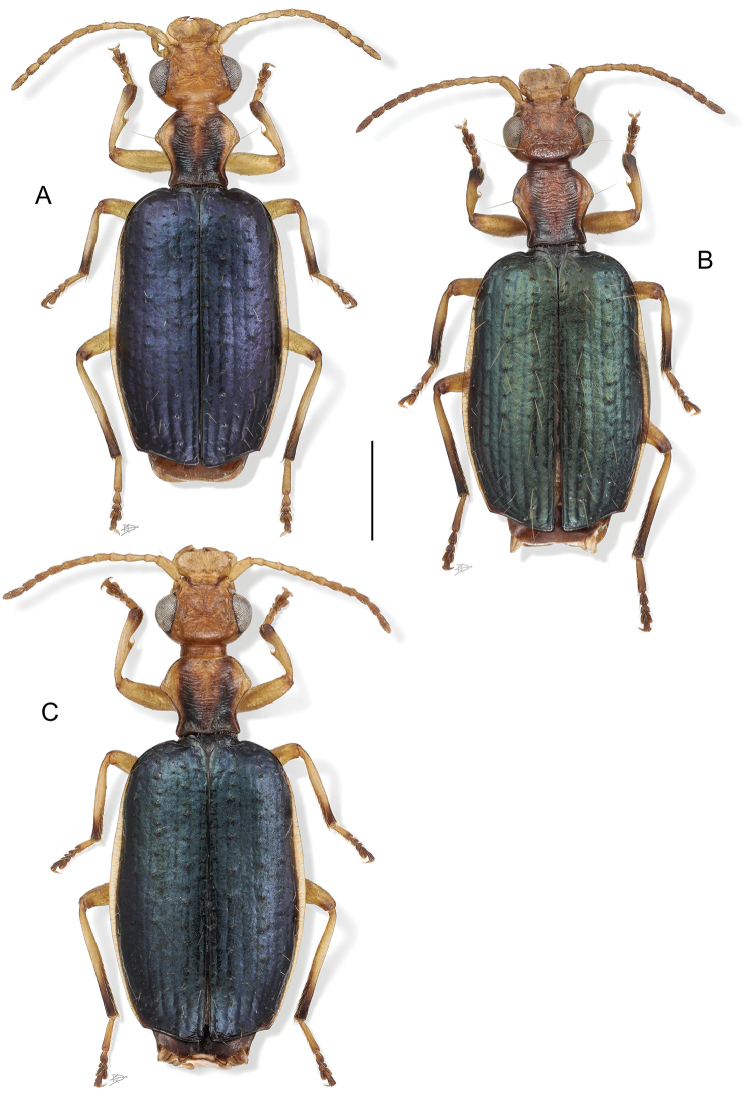
Digital Photo-illustrations. Habitus, dorsal aspect: **A** *Thoasia rugifrons* Liebke, female, ADP148165 **B** *Thoasia pterosmaragdos* Aldebron & Erwin, sp. n., female, ADP148174 **C** *Thoasia manu* Erwin & Aldebron, sp. n., female, ADP100320. Scale bar: 1.00 mm.

**Figure 2.**
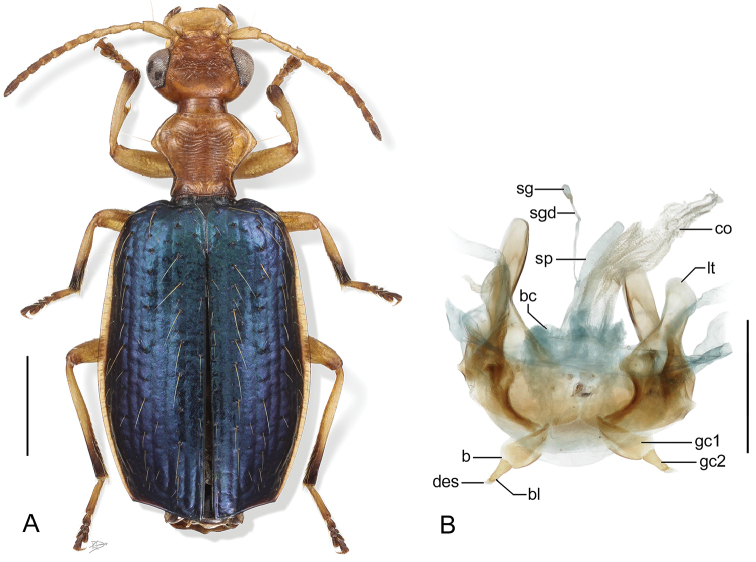
Digital Photo-illustrations. **A** Habitus, dorsal aspect: *Thoasia surinamensis* Erwin & Aldebron, sp. n., female, ADP112239. Scale line = 1.00 mm **B** Female reproductive system dorsal aspect: *Thoasia manu*, Erwin & Aldebron, sp. n., ADP100301. Legend, **bc**, bursa copulatrix; **sg**, spermathecal gland; **sgd**, spermathecal gland duct; **sp**., spermatheca; **lt**, laterotergite; **co**, common oviduct; **gc1**, gonocoxite 1; **gc2**, gonocoxite 2, **des**, dorsal ensiform seta, **b**, base of gonocoxite 2; **bl**, blade of gonocoxite 2. Scale bar: 0.50 mm.

**Figure 3.**
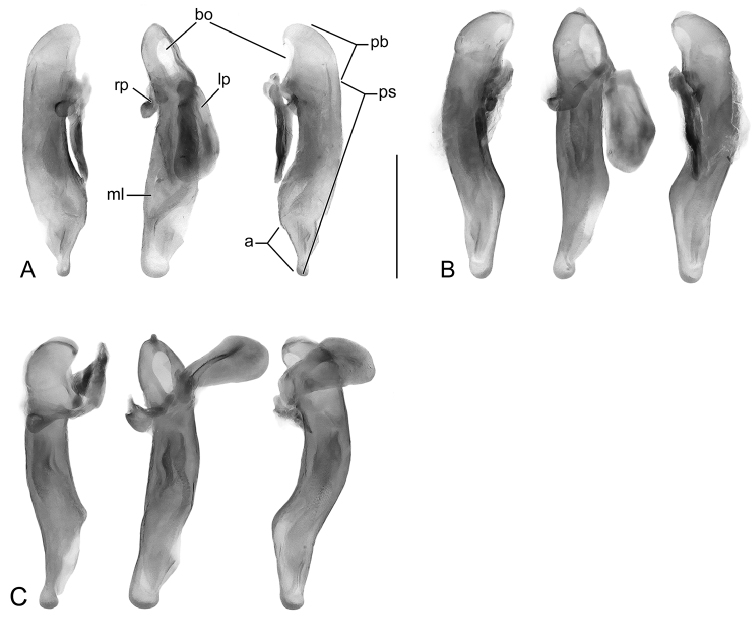
Digital Photo-illustrations, male aedeagus in repose, dorsal, ventral, left lateral aspects: **A** *Thoasia rugifrons* Liebke, ADP152522 **B** *Thoasia pterosmaragdos* Aldebron & Erwin, sp. n., ADP13748 **C** *Thoasia manu* Erwin & Aldebron, sp. n., ADP100336. Legend, **lp**, left paramere; **rp**, right paramere; **pb**, phallobase; **bo**, phallobase orifice; **ps**, phalloshaft; **a**, phalloapex; **ml**, median lobe. Scale bar: 0.50 mm.

**Figure 4.**
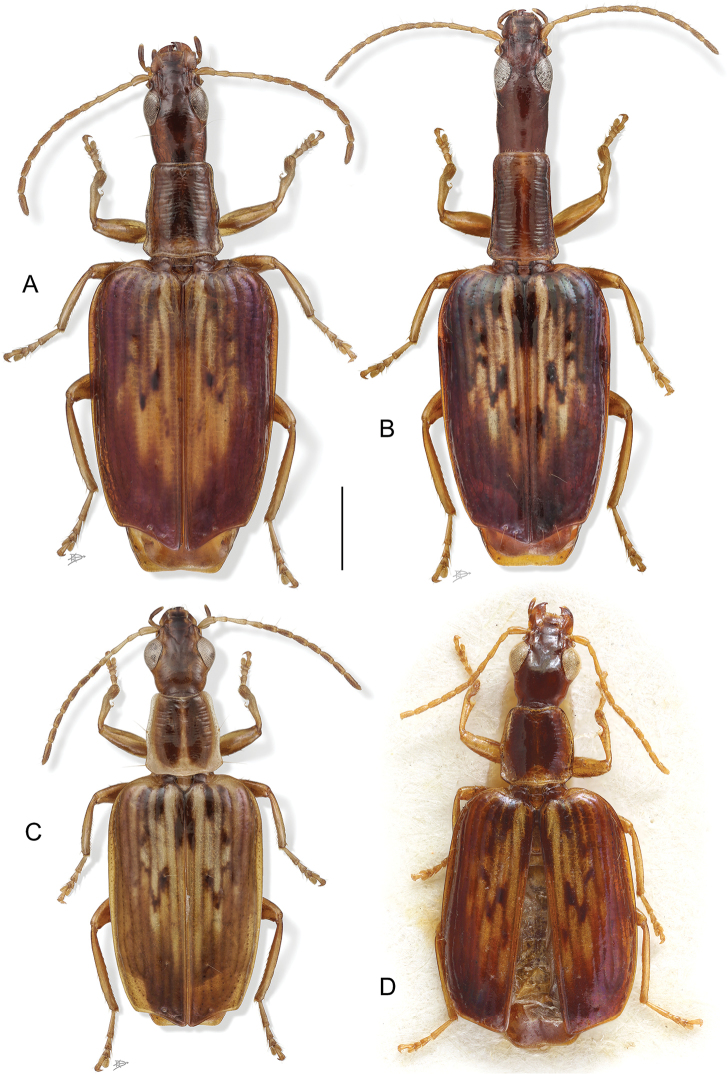
Digital Photo-illustrations. Habitus, dorsal aspect. **A** *Straneotia cylindroceps* Erwin & Aldebron, sp. n., female, ADP100396 **B** *Straneotia moi* Aldebron & Erwin, sp. n., female, ADP141236 **C** *Straneotia confundis* Aldebron & Erwin, sp. n., male, ADP152452 **D** *Straneotia amazonica* Mateu, Holotype female. Scale bar: 1.00 mm (**A–C**); ABL = 5.5mm (**D**).

Pentagonal arboreal carabid beetles Figs 1 , 2 , 3 , 4

Thoasia Liebke, 1939:129.

######## Type species.

*Thoasia rugifrons* Liebke 1939, by monotypy. Type area. **Venezuela**.

######## Diagnosis.

(cf. Figs 1, 2, 3, 4). Neck constricted, width less than that of base of pronotum; eyes moderately large, not hemispherical; frons moderately rugose, more or less anteriorly depressed. Prothorax more or less subequal in width to that of the head across eyes; sides of pronotum narrowly reflexed throughout, more broadly reflexed and markedly angulate at mid-lateral setigerous pore. Elytron at basal third not depressed, surface even, disc striate and punctate. Flight wings of a dusky color. Basitarsus of mid and hind legs markedly elongate, co-equal to length of tarsomeres 2-5; claws serrate. Male only with two rows of white adhesive setae on venter of tarsomeres 1-3; both sexes with long hooked white setae on tarsomere 5. Male with a pair of setae each side on abdominal sternum VII; female with 3 setae each side on abdominal sternum VII. Male endophallus without flagellum.

######## Dispersal potential.

The wings are fully developed in adults of all known species, thus it is likely these beetles are moderate to strong flyers.

######## Geographic distribution.

A widespread Neotropical genus known from Colombia, south to southeastern Brazil, in the west to Bolivia, and northeast to French Guiana.

######## Ways of life.

Little is known about the species in this genus and the little that is known is reported here for the first time. Adults of one species are regularly collected in both the wet and dry seasons using insecticidal fogging techniques in many species of trees reaching the forest canopy in the Amazon Basin, thus they are certainly most times arboreal. They are good flyers as evidenced by their capture in FITs in French Guiana.

######## Notes.

Only one species has been previously described in this genus from Venezuela and from only one specimen. Adults of the species most commonly collected in canopy samples in Ecuador and Perú were misidentified as *T. rugifrons* Liebke, but that species is only known to be from Venezuela and French Guiana. To date, the most common species in canopy samples is described below, as new.

######## References.

Liebke 1939; Erwin 1991.

######## Included species.

The species list below, as well as the arrangement of descriptions that follows, is ordered alphabetically within two species groups.

######### *rugifrons* species group

*Thoasia rugifrons* Liebke, 1939, French Guiana, Venezuela.

*Thoasia surinamensis* Erwin & Aldebron, **sp. n.**, Suriname.

*Thoasia pterosmaragdos* Aldebron & Erwin, **sp. n.**, French Guiana.

######### *manu* species group

*Thoasia manu* Erwin & Aldebron, **sp. n.**, Ecuador, Perú.

####### Key to the species of *Thoasia* Liebke, 1939

**Table:** 

1	Adults with two dark non-metallic stripes on the pronotal disc	**2**
1’	Adults without markings on the pronotum	***T. manu* , sp. n.**
2(1)	Adults with elytra dark blue	***T. rugifrons* Liebke**
2’	Adults with elytra pigmented otherwise	**3**
3(2)	Adults with elytra dark olivaceous	***T. surinamensis* , sp. n.**
3’	Adults with elytra emerald	***T. pterosmaragdos* , sp. n.**

####### *rugifrons* species group

The most distinctive attribute of species in this group is that the pronotum has two parallel dark stripes. Elytra of adults of all species have head and prothorax base coloration pale and elytra dark (metallic blue, green, or olivaceous) with mesothorax, metathorax, and abdomen black. Male phallus apex moderately elongate, bluntly rounded.

######## Thoasia rugifrons

Taxon classificationAnimaliaColeopteraCarabidae

Liebke, 1939

Rough-headed pentagonal arboreal carabid Figs 1A , 3A , 7

Thoasia rugifrons Liebke, 1939:129

######### Holotype.

**(sex unknown)**: Type area. **Venezuela**. Not seen by us; however, Mroczkowski (1960) indicates that the holotype is in the Polish Academy of Sciences in Warsaw. We have determined that Liebke’s translated description is good enough for us to recognize the species without seeing the type. Our redescription is based on newly acquired specimens (see below).

######### Derivation of specific epithet.

The epithet, *rugifrons*, is a Latinized singular feminine adjective meaning “rugose forehead.”

######### Proposed English Vernacular Name.

Rough-headed pentagonal arboreal carabid.

######### Diagnosis.

With the attributes of the genus and *rugifrons* species group as described above and adults with elytra metallic dark blue.

######### Description.

(Figs 1A, 3A). ***Habitus***: (Fig. 1A). *Size*: See Appendix 1. Length (SBL) long for genus, ABL = 4.30–4.55 mm, SBL = 4.11–4.16 mm.

***Color***: *See* diagnosis above. ***Luster***: Elytra shiny metallic; forebody and head shiny. ***Microsculpture***: Mostly isodiametric, well-impressed. ***Head***: Rugae moderately coarse, mostly transverse on occiput, longitudinal adjacent to eye, slightly angulate on frons. Eye very large, sub-hemispheric, and evenly rounded anteriorly, subtly more prolonged posteriorly. Antenna moderately long, reaching humerus. Labrum very large, somewhat cordate, shallowly convex at middle. Neck constricted, width coequal to anterior margin of pronotum. ***Prothorax***: Pronotum moderately narrow, disk centrally convex, with dense transverse rugae and two dark stripes extending longitudinally, occasionally converging at the base. Lateral margins at apical third explanate and sharply acute, becoming constricted at basal third. Hind angles moderately acute and subtly narrower than lateral margin at widest point. ***Pterothorax***: Normal for Agrina, fully winged, wings smoky translucent. Elytron intervals 3, 5, and 7 with numerous discal setae, intervals slightly convex, side margin markedly explanate. Elytron narrow and short, moderately wider than the pronotum at the broadest part, apex truncate, slightly sinuate with distal corner obtusely rounded, disc not significantly convex, basal third not depressed. All interneurs well-impressed. ***Legs***: Normal for Agrina, no unique modifications. ***Abdomen***: Sparsely setiferous; normal ambulatory setae on sterna 3–5; female with three pairs of ambulatory setae on sternum 6; males with two pairs of longer setae on sternum 6. ***Male genitalia***: Phallus (Fig. 3A.) broad with phallobase ⅓ of its length, ostium 1/3 of its length, catopic, apex short, markedly broad and shallowly rounded; endophallus moderately complex without flagellum. Parameres asymmetric, right very small, left larger, broad, apically rounded. ***Female genitalia***: (cf. Fig. 2B). Unstudied, but likely similar to *T. manu*, new species.

######### Dispersal potential.

These beetles are macropterous and capable of flight. They are likely moderately swift and agile runners. The two specimens were collected in flight intercept traps (FITs), one located at ground level and the other at 20 m up in the canopy.

######### Way of life.

The specimens were collected in January and February, where the climate is tropical, hot and humid throughout the year, with a relatively dry and slightly warmer season from July to November, and a rainy season from December to June.

######### Other specimens examined.

**French Guiana**: Cayenne, Commune de Roura, RN2, PK22, Montagne des Chevaux, 4.7127°N, 52.3966°W, 90 m, 12 February 2012 (S. Brûlé, PH. Dalens, E. Poirier)(NMNH: ADP152522, male), 9 January 2016 (S. Brûlé, PH. Dalens, E. Poirier)(NMNH: ADP148165, female).

######### Geographic distribution.

(Fig. 7). This species is currently known from the type locality in Venezuela and in the lowlands of French Guiana.

######### Note.

In Liebke’s description, his specimen measures 5.5 mm. This would mean that the range of ABL for *T. rugifrons* is greater than one millimeter, a significant enough variation both within this and among other species of this genus to warrant the consideration of error in measurement on his part.

######## Thoasia surinamensis

Taxon classificationAnimaliaColeopteraCarabidae

Erwin & Aldebron sp. n.

http://zoobank.org/C1C875CB-BFC5-405B-ABB1-F8C88FF3C80E

Suriname pentagonal arboreal carabid Figs 2A , 7

######### Holotype.

(Female): Type locality. **Suriname**, Saramacca, 5.8177°N, 55.5906°W, 15–25 September 2005 (G. J. Steck)(FSCA: ADP112239).

######### Derivation of specific epithet.

The epithet, *surinamensis*, is a Latinized noun referring the type locality area of the species.

######### Proposed English Vernacular Name.

Suriname pentagonal arboreal carabid.

######### Diagnosis.

With the attributes of the genus and *rugifrons* species group as described above and adults with unicolorous ferrugineus head and pronotum, the latter with two longitudinal infuscated stripes from apex to base, elytra entirely dark olivaceous except translucent, narrowly explanate lateral margin, scutellum, meso-, and metathorax, and abdomen black, tibial and femoral apex infuscated.

######### Description.

(Fig. 2A). ***Habitus***: (Fig. 2A). *Size*: See Appendix 1. Length (SBL) average for genus, ABL = 4.72 mm, SBL = 4.12 mm.

***Color***: *See* diagnosis above, and distal antennomers slightly infuscated. ***Luster***: Elytra shiny olivaceous; forebody and head shiny. ***Microsculpture***: Mostly isodiametric, well-impressed. ***Head***: Rugae moderately coarse, mostly transverse on fore-occiput, longitudinal on aft-occiput and adjacent to eye, slightly angulate on frons. Eye large, sub-hemispheric, and evenly rounded anteriorly, subtly more prolonged posteriorly. Antenna moderately long, reaching humerus. Labrum very large, slightly cordate, shallowly convex at middle. Neck constricted, width coequal to anterior margin of pronotum. ***Prothorax***: Pronotum moderately narrow, disc centrally convex with dense transverse rugae. Lateral margins at middle explanate and sharply acute then markedly arcuate to obtusely lobed hind angle; base subtly produced medially. ***Pterothorax***: Normal for Agrina, fully winged, wings smoky translucent. Elytron intervals 3, 5, and 7 with numerous long discal setae, intervals slightly convex, side margin moderately explanate. Elytron moderately broad and short, moderately wider than the pronotum at the broadest part, apex truncate, slightly sinuate with distal corner obtusely rounded, disc not significantly convex, basal third not depressed. All interneurs well-impressed. ***Legs***: Normal for Agrina, no unique modifications. ***Abdomen***: Sparsely setiferous; normal ambulatory setae on sterna 3–5; female with three pairs of ambulatory setae on sternum 6; male unknown but probably with two pairs of longer setae on sternum 6, as in *T. manu*. ***Male genitalia***: Unknown. ***Female genitalia***: (cf. Fig. 2B). Unstudied, but likely similar to *T. manu*.

######### Dispersal potential.

These beetles are macropterous and capable of flight. They are probably moderately swift and agile runners. The single specimen was collected in a malaise trap suspended at 6m up.

######### Way of life.

The holotype was active in September, the cooler minor wet season in Suriname.

######### Other specimens examined.

None.

######### Geographic distribution.

(Fig. 7). This species is currently known from the type locality in Suriname.

######## Thoasia pterosmaragdos

Taxon classificationAnimaliaColeopteraCarabidae

Aldebron & Erwin sp. n.

http://zoobank.org/60AAC432-E879-4C24-ACD5-9B4018CFE19F

Emerald-winged pentagonal arboreal carabid Figs 1B , 3B , 7

######### Holotype.

(Female): Type locality. **French Guiana**, Saint-Laurent-du-Maroni, Commune de Regina, Nouragues, Camp Inselberg, 4.0833°N, 52.6833°W, 13 October 2013 (S. Brûlé, PH. Dalens, E. Poirier)(NMNH: ADP148174).

######### Derivation of specific epithet.

The epithet, *pterosmaragdos*, is an ancient Greek derivation comprised of the words “ptero,” meaning wing, and “smaragdos,” meaning emerald. It refers to the emerald green hue of the elytra.

######### Proposed English Vernacular Name.

Emerald-winged pentagonal arboreal carabid.

######### Diagnosis.

With the attributes of the genus and *rugifrons* species group as described above and adults with unicolorous ferrugineus head and pronotum, elytra entirely metallic dark emerald except translucent, explanate, narrow lateral margin, and meso-, and metathorax and abdomen black. Aedeagus narrow with apex narrow, twisted.

######### Description.

(Figs 1B, 3B). ***Habitus***: (Fig. 1B). *Size*: See Appendix 1. Length (SBL) average for genus, ABL = 4.97–5.11 mm, SBL = 4.21–4.39 mm.

***Color***: *See* diagnosis above. ***Luster***: Elytra shiny, metallic. ***Microsculpture***: Mostly isodiametric, well-impressed. ***Head***: Frons with rugous depressions, bisected by centrally produced carina, perpendicular to clypeus, occiput with transverse, moderately coarse rugae. Eye large, sub-hemispheric, and evenly rounded anteriorly, subtly more prolonged posteriorly. Antenna moderately long, reaching humerus. Labrum very large, somewhat cordate, shallowly convex at middle. Neck constricted, width coequal to anterior margin of pronotum. ***Prothorax***: Pronotum moderately narrow, disk centrally convex, with dense transverse rugae and two dark stripes extending longitudinally, occasionally converging at the base. Lateral margins at apical third explanate and sharply acute, becoming constricted at basal third. Hind angles moderately acute. Differs from *T. rugifrons* in that lateral angles are markedly broader than hind angles. ***Pterothorax***: Normal for Agrina, fully winged, wings dark emerald in color. Elytron intervals 3, 5, and 7 with numerous discal setae, intervals slightly convex, side margin markedly explanate. Elytron moderately narrow and short, moderately wider than the pronotum at the broadest part, apex truncate, slightly sinuate with distal corner obtusely rounded, disc not significantly convex, basal third not depressed. All interneurs well-impressed. ***Legs***: Normal for Agrina. Apex of femur dusty brown. ***Abdomen***: Sparsely setiferous; normal ambulatory setae on sterna 3–5; female with three pairs of ambulatory setae on sternum 6; males with two pairs of longer setae on sternum 6. ***Male genitalia***: Phallus (Fig. 3B) narrow, phallobase short, ostium 1/3 of its length, catopic, apex narrow, longitudinally twisted, moderately rounded; endophallus moderately complex without flagellum. Parameres asymmetric, right very small, left larger, broad, apically rounded. ***Female genitalia***: (cf. Fig. 2B) Unstudied, but likely similar to *T. manu*.

######### Dispersal potential.

These beetles are macropterous and capable of flight, as evidenced by collection through flight intercept traps.

######### Way of life.

Specimens where the specimens were collected in February, September, and October, the climate is tropical, hot and humid throughout the year, with a relatively dry and slightly warmer season from July to November, and a rainy season from December to June.

######### Other specimens examined.

**French Guiana**: Saint-Laurent-du-Maroni, Commune de Saül, Belvedere de Saül, 3.6223°N, 53.2159°W, 283–325 m, 17 February 2010 (S Brûlé, PH Dalens, E Poirier)(NMNH: ADP130748, male paratype); Cayenne, Commune de Regina, RN2, PK65, Cirque Orfion Orapu, 4.4961°N, 52.3453°W, 17 September 2016 (S Brûlé, PH Dalens, E Poirier)(NMNH: ADP151211, male paratype).

######### Geographic distribution.

(Fig. 7). This species is currently known from three localities in French Guiana.

####### *manu* species group

The most distinctive attribute of species in this group is that the pronotum is unicolorous with no parallel dark stripes. Elytra of adults have metallic blue coloration. Male phallus apex short, broadly rounded.

######## Thoasia manu

Taxon classificationAnimaliaColeopteraCarabidae

Erwin & Aldebron sp. n.

http://zoobank.org/C613C9FA-AF2C-48AD-A132-1F7775E75484

Río Manu pentagonal arboreal carabid Figs 1C , 2B , 3C , 7

######### Holotype.

(Female): Type locality. **Perú**, Madre de Dios, Manu Reserved Zone, Río Manu, BIOLAT Biological Station, Pakitza, 11.9446°S, 71.2831°W, 356m, 16 October 1989 (TL Erwin)(NMNH, ADP100320).

######### Derivation of specific epithet.

The epithet, *manu*, is a singular feminine noun referring to the type locality of the species, the Río Manú, in southeastern Perú.

######### Proposed English Vernacular Name.

Río Manú pentagonal arboreal carabid.

######### Diagnosis.

With the attributes of the genus and *manu* species group as described above and adults with unicolorous head and pronotum, elytra entirely metallic blue except translucent narrow lateral margin, and meso-, metathorax and abdomen black.

######### Description.

(Figs 1C, 2B, 3C). ***Habitus***: (Fig. 1C). *Size*: See Appendix 1. Length (SBL) average for genus, ABL = 4.42–5.22 mm, SBL = 3.91–4.77.

***Color***: *See* diagnosis above. ***Luster***: Elytra shiny metallic; forebody and head shiny flavous. ***Microsculpture***: Mostly isodiametric, well-impressed. ***Head***: Rugae moderately coarse, mostly transverse on occiput, longitudinal adjacent to eye, slightly angulate on frons. Eye very large, sub-hemispheric, and evenly rounded anteriorly, subtly more prolonged posteriorly. Antenna moderately long, reaching humerus. Labrum very large, somewhat cordate, shallowly convex at middle. Neck constricted, width coequal to anterior margin of pronotum. ***Prothorax***: Pronotum moderately narrow, disc centrally convex with dense transverse rugae. Lateral margins at middle explanate and sharply acute then markedly arcuate to obtusely lobed hind angle; base subtly produced medially. ***Pterothorax***: Normal for Agrina, fully winged, wings smoky translucent. Elytron intervals 3, 5, and 7 with numerous discal setae, intervals slightly convex, side margin markedly explanate. Elytron moderately broad and short, moderately wider than the pronotum at the broadest part, apex truncate, slightly sinuate with distal corner obtusely rounded, disc not significantly convex, basal third not depressed. All interneurs well-impressed. ***Legs***: Normal for Agrina, no unique modifications. ***Abdomen***: Sparsely setiferous; normal ambulatory setae on sterna 3–5; female with three pairs of ambulatory setae on sternum 6; males with two pairs of longer setae on sternum 6. ***Male genitalia***: Phallus (Fig. 3C) with ostium of 1/3 its length, catopic, apex short, broadly and shallowly rounded; endophallus moderately complex without flagellum. Parameres asymmetric, right very small, left larger, narrow, apically rounded. ***Female genitalia***: (Fig. 2B). Ovipositor with broad spatulate laterotergite (lt) and two gonocoxites (gc 1, gc 2); gonocoxite 1 robust and apicolaterally asetose; gonocoxite 2 apically subacuminate, base (b) medium-size broader in width than blade (bl) which is moderately elongate and with several short apical ensiform setae (des), without ventral preapical nematiform setae. Reproductive tract proximally with moderately long, broad bursa copulatrix (bc), continuous at its distal end with common oviduct and long robust corrugated spermatheca (sp) distal to villous canal; spermathecal gland small, bulbous; spermathecal gland duct (sgd) very slim, attached to oviduct at base of its broadened portion.

######### Variation.

Given the sampling of specimens we have for this group, we were able to note a variation in size corresponding with locality. Specimens from Ecuador are larger (SBL: 4.7–5.07 mm, TW: 1.94–2.33 mm) than those from Perú (SBL: 3.91–4.68 mm, TW: 1.83–2.26 mm). After a comparison of external attributes supported by identical male genitalia, we have determined them to be conspecific.

######### Dispersal potential.

These beetles are macropterous and probably capable of flight. They are probably moderately swift and agile runners. They have only been collected using fogging techniques.

######### Way of life.

Adults are common in the western Amazonian lowlands (100 to 400 m.a.s.l.) and appear to be generalists in a variety of rainforest biotopes including terra firme, várzea, and igapó. In these forests, they are commonly found in big trees such as species of *Guarea*, *Virola*, *Spondias*, *Quararibea* with vines and epiphytes, and in suspended dry leaves, in dry fronds of *Astrocaryum chambira* Burret, *Maurita flexuosa*, *Jessenia bataua*, *Iriartea deltoidea* and *Scheelea* sp. and in dry bamboo leaves of the species *Guadua weberbaueri* Pilg. Individuals are found in most months of the year, during both the rainy and dry seasons. Members of this species have been recorded from the canopy of the aforementioned genera and other vegetation noted below using insecticidal fogging techniques. The following are the trees, vines, and palms that botanists have identified (with their notations) in our fogging plot at the Onkone Gare Camp, Reserva Etnica Waorani: *Lacistema nena* cf.; *Guatteria glaberrima* cf.; *Protium sagotianum* cf.; *Guatteria* sp. 3, sect.; *Meiocarpus*, long petiole; *Oenocarpus bataua*; *Neea* “dive-tuberculate”; *Semaphyllanthe megistocaula* cf.; Lauraceae “redvein”; *Eschweilera coriacea* cf.; *Protium* “sect. *Icicopsis*” sp. nov. ined.; *Iriartea deltoidea*; *Grias neuberthii*; *Brownea grandiceps* cf.; *Talisia* “bitter”; *Virola decorticans* cf.; Pouteria cuspidata ssp. robusta cf.; *Virola obovata*; Brosimum utile ssp. ovatifolium cf.; *Inga auristellae* cf.; *Compsoneura capitellata* cf.; *Coccoloba densifrons*; *Gustavia longifolia*; *Browneopsis ucayalina*; Annonaceae; *Leonia glycicarpa*; *Protium subserratum*; *Wettinia maynensis*; *Capirona*; *Pouteria baehniana* cf.; Pourouma bicolor ssp. bicolor cf.; *Licania britteniana* “rounded”; *Virola flexuosa*; *Sarcaulus brasiliensis* aff. “burnt”; *Tetrathylacium macrophyllum*; *Trymatococcus amazonicus* cf.; *Chrysophyllum argenteum* cf.; *Sterculia colombiana* cf.; *Parkia multijuga* cf.; *Naucleopsis herrerensis* cf.; *Matisia malacocalyx* cf.; *Pseudolmedia laevigata*; *Mauritia flexuosa*.

######### Other specimens examined.

**Ecuador**: Orellana, 43 km S, Pompeya, Maxus Road, Palm Swamp, Reserva Etnica Waorani, 0.6850°S, 76.4321°W, 233m, 3 July 1996 (T.L. Erwin, et al.)(NMNH: ADP149900, male paratype), Estación Científica Yasuní - Onkone Gare Camp, 39 km S, Pompeya, Erwin Piraña Plot: t-2 sta. 1, Reserva Etnica Waorani, 0.6581°S, 76.4513°W, 220-250m, 22 January 1994 (T.L. Erwin, et al.)(NMNH: ADP137916, female paratype), Plot: t-8 sta. 1, Reserva Etnica Waorani, 0.6551°S, 76.4403°W, 220-250m, 21 June 1994 (T.L. Erwin, et al.)(NMNH: ADP138371, female paratype), Plot: t-10 sta. 8, Reserva Etnica Waorani, 0.6540°S, 76.4453°W, 220-250m, 6 October 1994 (T.L. Erwin, et al.)(NMNH: ADP137701, male paratype), Plot: t-8 sta. 7, Reserva Etnica Waorani, 0.6551°S, 76.4403°W, 220-250m, 7 October 1994 (T.L. Erwin, et al.)(NMNH: ADP100300, female paratype), Plot: t-8 sta. 7, Reserva Etnica Waorani, 0.6551°S, 76.4521°W, 220-250m, 7 October 1994 (T.L. Erwin, et al.)(NMNH: ADP100300, female paratype), Plot: t-3 sta. 5, Reserva Etnica Waorani, 0.6575°S, 76.4505°W, 220-250m, 10 October 1994 (T.L. Erwin, et al.)(NMNH: ADP136284, female paratype), Plot: t-10 sta. 3, Reserva Etnica Waorani, 0.6540°S, 76.4453°W, 220-250m, 8 February 1995 (T.L. Erwin, et al.)(NMNH: ADP137686, female paratype), Plot: t-10 sta. 6, Reserva Etnica Waorani, 0.6540°S, 76.4453°W, 220-250m, 8 February 1995 (T.L. Erwin, et al.)(NMNH: ADP137615, female paratype), Plot: t-3 sta. 10, Reserva Etnica Waorani, 0.6575°S, 76.4505°W, 220-250m, 1 July 1995 (T.L. Erwin, et al.)(NMNH: ADP138769, male paratype), Plot: t-6 sta. 10, Reserva Etnica Waorani, 0.6561°S, 76.4487°W, 220-250m, 7 February 1996 (T.L. Erwin, et al.)(NMNH: ADP137625, male paratype), Plot: t-7 sta. 8, Reserva Etnica Waorani, 0.6551°S, 76.4403°W, 220-250m, 8 February 1996 (T.L. Erwin, et al.)(NMNH: ADP138217, female paratype), Plot: t-8 sta. 10, Reserva Etnica Waorani, 0.6551°S, 76.4403°W, 220-250m, 8 February 1996 (T.L. Erwin, et al.)(NMNH: ADP138261, male paratype), Plot: t-8 sta. 7, Reserva Etnica Waorani, 0.6551°S, 76.4403°W, 220-250m, 8 February 1996 (T.L. Erwin, et al.)(NMNH: ADP138237, female paratype), Plot: t-9 sta. 1, Reserva Etnica Waorani, 0.6545°S, 76.4460°W, 220-250m, 23 June 1996 (T.L. Erwin, et al.)(NMNH: ADP136330, female paratype), Plot: t-9 sta. 1, Reserva Etnica Waorani, 0.6545°S, 76.4460°W, 220-250m, 23 June 1996 (T.L. Erwin, et al.)(NMNH: ADP136340, female paratype), Plot: t-2 sta. 9, Reserva Etnica Waorani, 0.6581°S, 76.4513°W, 220-250m, 25 June 1996 (T.L. Erwin, et al.)(NMNH: ADP138072, male paratype), Plot: t-2 sta. 9, Reserva Etnica Waorani, 0.6581°S, 76.4513°W, 220-250m, 25 June 1996 (T.L. Erwin, et al.)(NMNH: ADP138181, male paratype), Plot: t-8 sta. 3, Reserva Etnica Waorani, 0.6551°S, 76.4403°W, 220-250m, 3 October 1996 (T.L. Erwin, et al.)(NMNH: ADP137730, male paratype), Plot: t-8 sta. 5, Reserva Etnica Waorani, 0.6551°S, 76.4403°W, 220-250m, 3 October 1996 (T.L. Erwin, et al.)(NMNH: ADP137914, male paratype), Plot: t-2 sta. 10, Reserva Etnica Waorani, 0.6581°S, 76.4513°W, 220-250m, 4 October 1995 (T.L. Erwin, et al.)(NMNH: ADP136343, female paratype), Plot: t-6 sta. 7, Reserva Etnica Waorani, 0.6561°S, 76.4483°W, 220-250m, 6 October 1995 (T.L. Erwin, et al.)(NMNH: ADP138547, female paratype), Plot: t-10 sta. 7, Reserva Etnica Waorani, 0.6540°S, 76.4453°W, 220-250m, 8 October 1995 (T.L. Erwin, et al.)(NMNH: ADP138888, male paratype), Plot: t-1 sta. 4, Reserva Etnica Waorani, 0.6586°S, 76.4521°W, 220-250m, 30 January 2006 (T.L. Erwin, et al.)(NMNH: ADP143309, female paratype), Yasuní National Park (edge), 95.43 km E (heading 101.46°) , Coca, Erwin Harpia Plot: t-4 sta. 4, Estación de Biodiversidad Tiputini, 0.6316°S, 76.1443°W, 208m, 30 June 1998 (T.L. Erwin, et al.)(NMNH: ADP137461, female paratype), Plot: t-4 sta. 5, Estación de Biodiversidad Tiputini, 0.6316°S, 76.1443°W, 208m, 24 October 1998 (T.L. Erwin, et al.)(NMNH: ADP136691, female paratype), Plot: t-9 sta. 6, Estación de Biodiversidad Tiputini, 0.6269°S, 76.1443°W, 203m, 5 February 1999 (T.L. Erwin, et al.)(NMNH: ADP136843, female paratype), Plot: t-6 sta. 1, Estación de Biodiversidad Tiputini, 0.6295°S, 76.1443°W, 199m, 7 February 1999 (T.L. Erwin, et al.)(NMNH: ADP136850, female paratype), Plot: t-4 sta. 4, Estación de Biodiversidad Tiputini, 0.6316°S, 76.1443°W, 203m, 8 February 1999 (T.L. Erwin, et al.)(NMNH: ADP136821, male paratype), Plot: t-4 sta. 4, Estación de Biodiversidad Tiputini, 0.6316°S, 76.1443°W, 203m, 8 February 1999 (T.L. Erwin, et al.)(NMNH: ADP136823, female paratype), Plot: t-4 sta. 4, Estación de Biodiversidad Tiputini, 0.6316°S, 76.1443°W, 203m, 8 February 1999 (T.L. Erwin, et al.)(NMNH: ADP136827, male paratype), Plot: t-4 sta. 4, Estación de Biodiversidad Tiputini, 0.6269°S, 76.1443°W, 203m, 23 October 1999 (T.L. Erwin, et al.)(NMNH: ADP139273, male paratype), Plot: t-6 sta. 10, Estación de Biodiversidad Tiputini, 0.6295°S, 76.1443°W, 199m, 25 October 1999 (T.L. Erwin, et al.)(NMNH: ADP143014, male paratype), Plot: t-9 sta. 6, Estación de Biodiversidad Tiputini, 0.6269°S, 76.1443°W, 203m, 28 October 1999 (T.L. Erwin, et al.)(NMNH: ADP143137, male paratype), Plot: t-9 sta. 6, Estación de Biodiversidad Tiputini, 0.6269°S, 76.1443°W, 203m, 17 February 2001 (T.L. Erwin, et al.)(NMNH: ADP143417, male paratype), Plot: t-9 sta. 6, Estación de Biodiversidad Tiputini, 0.6269°S, 76.1443°W, 203m, 17 February 2001 (T.L. Erwin, et al.)(NMNH: ADP143425, female paratype), Plot: t-7 sta. 10, Estación de Biodiversidad Tiputini, 0.6284°S, 76.1443°W, 203m, 18 February 2001 (T.L. Erwin, et al.)(NMNH: ADP143495, male paratype), Plot: t-6 sta. 9, Estación de Biodiversidad Tiputini, 0.6295°S, 76.1443°W, 199m, 19 February 2001 (T.L. Erwin, et al.)(NMNH: ADP139426, female paratype), Plot: t-9 sta. 6, Estación de Biodiversidad Tiputini, 0.6269°S, 76.1443°W, 203m, 20 July 2001 (T.L. Erwin, et al.)(NMNH: ADP143434, male paratype), Plot: t-9 sta. 6, Estación de Biodiversidad Tiputini, 0.6269°S, 76.1443°W, 203m, 20 July 2001 (T.L. Erwin, et al.)(NMNH: ADP143438, female paratype), Plot: t-9 sta. 7, Estación de Biodiversidad Tiputini, 0.6269°S, 76.1443°W, 203m, 17 February 2001 (T.L. Erwin, et al.)(NMNH: ADP142443, male paratype), Plot: t-9 sta. 9, Estación de Biodiversidad Tiputini, 0.6269°S, 76.1443°W, 203m, 17 February 2001 (T.L. Erwin, et al.)(NMNH: ADP143485, female paratype), **Perú**: Loreto, Camp Manco Capac, Río Samiria, 4.8722°S, 74.3565°W, 106m, 30 May 1990 (T.L. Erwin, et al.)(NMNH: ADP092186, male paratype, female paratype), 24 June 1990 (T.L. Erwin, et al.)(NMNH: ADP066785, ADP093715, ADP093722, female paratypes), Camp Boca del Inglés, 1 km E, Hamburgo, Pacaya-Samiria National Reserve, 5.6951°S, 75.1833°W , 150m, 10 May 1990 (T.L. Erwin, et al.)(NMNH: ADP100369, ADP100368, male paratypes), Camp Terry, Pacaya-Samiria National Reserve, 5.6951°S, 75.2243°W, 129m, 14 May 1990 (T.L. Erwin)(NMNH: ADP093075, female paratype), Cocha Shinguito, Pacaya-Samiria National Reserve, 5.1768°S, 74.6554°W, 111m, 24 May 1990, T.L. Erwin, et al.)(NMNH: ADP067076, ADP067138, female paratypes), 24 May 1990, T.L. Erwin, M.G. Pogue, et al.)(NMNH: ADP067072, ADP067099, ADP067117, male paratypes), 27 August 1991, T.L. Erwin, M.G. Pogue, et al.)(NMNH: ADP050361, male paratype, ADP071162, female paratype), ACEER-Explornapo Camp, Río Sucusari, Río Napo, 3.2591°S, 72.9163°W, 101m, 15 June 1992, T.L. Erwin, E Pfuno, F. Pfuno, NMNH: ADP052710, female paratype); Madre de Dios, BIOLAT Biological Station, Río Manu, Pakitza, Manu Reserved Zone, 11.9440°S, 71.2830°W, 356m, 26 September 1991 (T.L. Erwin)(NMNH: ADP100346, male paratype), 2 October 1991 (T.L. Erwin, M.G. Pogue)(NMNH: ADP100348, male paratype), 6 October 1991 (T.L. Erwin, M.G. Pogue)(NMNH: ADP100335, male paratype), Madre de Dios, BIOLAT Biological Station, Río Manu, Pakitza, Manu Reserved Zone, 11.9440°S, 71.2830°W, 356m, 6 October 1991 (T.L. Erwin, M.G. Pogue)(NMNH: ADP100347, female paratype), 9 October 1991 (T.L. Erwin, M.G. Pogue)(NMNH: ADP100325, ADP100330, ADP100331, ADP100326, ADP100336, ADP100338, male paratypes; ADP100329, ADP100332, ADP100333, ADP100334, ADP100337, female paratypes), 10 October 1991 (T.L. Erwin, M.G. Pogue)(NMNH: ADP100321, ADP100355, ADP100357, male paratypes; ADP100294, ADP100322, ADP100323, ADP100356, ADP100358, female paratypes), 14 October 1991 (T.L. Erwin, M.G. Pogue)(NMNH: ADP100352, ADP100353, male paratypes; ADP100315, ADP100350, ADP100351, ADP100354, female paratypes), 16 October 1991 (M.G. Pogue)(NMNH: ADP100340, ADP100342, ADP100349, ADP100298 ADP100309, ADP100311, ADP100312, ADP100316, ADP100318, ADP100319, ADP100324, ADP100327, ADP100345, male paratypes; ADP100339, ADP100320, ADP100317, ADP100328, ADP100313, ADP100314, ADP100343, ADP100307, ADP100308, ADP100344, female paratypes), 22 June 1993 (T.L. Erwin, F. Pfuno)(NMNH: ADP100293, male paratype; ADP100295, ADP100296, ADP100299, ADP100297, female paratypes), 11 September 1988 (T.L. Erwin, B.D. Farrell)(NMNH: ADP100306, male paratype; ADP100301, female paratype), 2 September 1989)(T.L. Erwin, B.D. Farrell, NMNH: ADP100302, male paratype), 18 September 1989 (T.L. Erwin, B.D. Farrell)(NMNH: ADP100304, male paratype; ADP100303, ADP100305, female paratypes), 16 March 1982 (T.L. Erwin, et al.)(NMNH: ADP100360, male paratype; ADP100359, female paratype), 22 March 1982 (T.L. Erwin, et al.)(NMNH: ADP100361, female paratype), Explorer’s Inn, 30km (air) SW, Pto. Maldonado, Reserva Río Tambopata, 12.8364°S, 69.2938°W, 208m, 10 May 1984 (T.L. Erwin, et al.)(NMNH: ADP100363, ADP100364, male paratypes; ADP149902, female paratype), 10 September 1984 (T.L. Erwin, et al.)(NMNH: ADP100366, female paratype), 8 November 1983 (T.L. Erwin, et al.)(NMNH: ADP100365, male paratype), 12 November 1983 (T.L. Erwin, et al.)(NMNH: ADP100367, female paratype).

######### Geographic distribution.

(Fig. 7). This species is currently known from the type locality in the Amazonian lowlands and at several other localities in Perú, as well as in the Yasuní region of Ecuador.

######### Notes.

The holotype is held in trust at NMNH until the completion of our Cryptobatida Group studies and then will be deposited in the Museum at the Universidad Nacional Mayor de San Marcos (MUSM), Lima, Perú.

###### Revision of the genus *Straneotia* Mateu, 1961

####### Straneotia

Taxon classificationAnimaliaColeopteraCarabidae

Mateu, 1961

**Figure 5.**
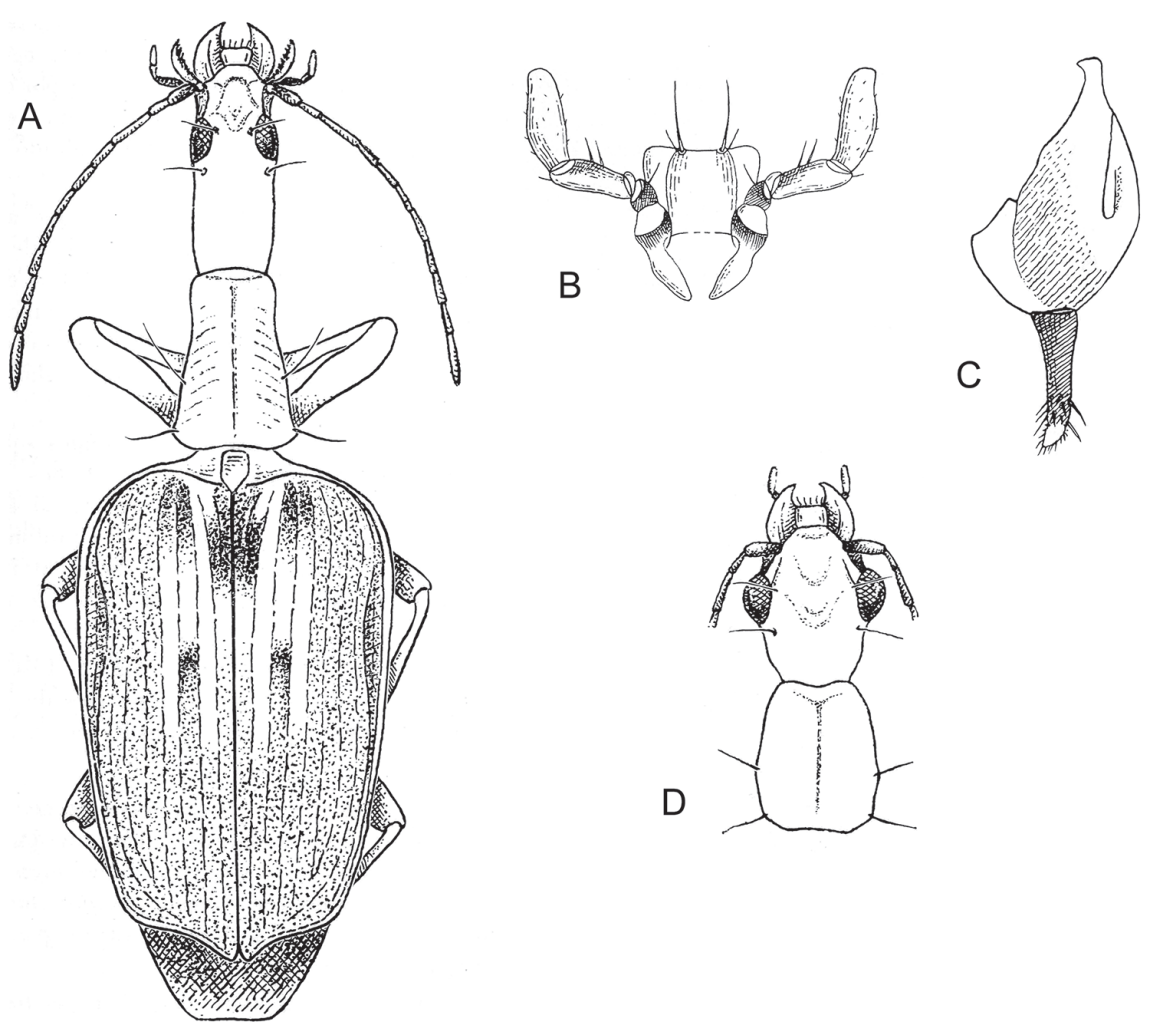
Illustrations from the orginal descriptions by Mateu (1961). Habitus, dorsal aspect. **A** *Straneotia freyi* Mateu, female; Ligula and labial palpi, ventral aspect **B** *Straneotia freyi* Mateu, female **C** Stylomeres 1 and 2, dorsal aspect. *Straneotia freyi* Mateu, female **D** Head and pronotum, dorsal aspect. *Straneotia amazonica* Mateu, female.

**Figure 6.**
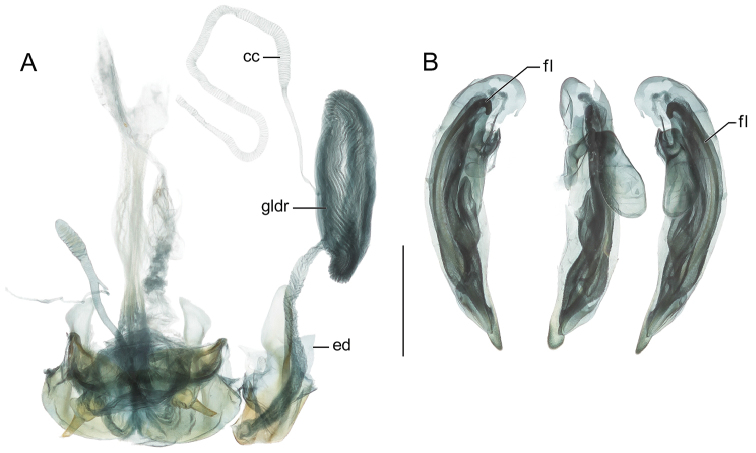
Digital Photo-illustrations. **A** Female reproductive and defence systems, dorsal aspect: *Straneotia cylindroceps* Erwin & Aldebron, sp. n., ADP100396 (see Fig. 2 references to female system; defense gland (**gldr**); **cc** accessory gland; **ed** efferent duct.). **B** Male aedeagus in repose, dorsal, ventral, left lateral aspects: *Straneotia confundis* Aldebron & Erwin, sp. n., ADP152452. Legend, fl, flagellum. Scale bar: 0.50 mm.

**Figure 7.**
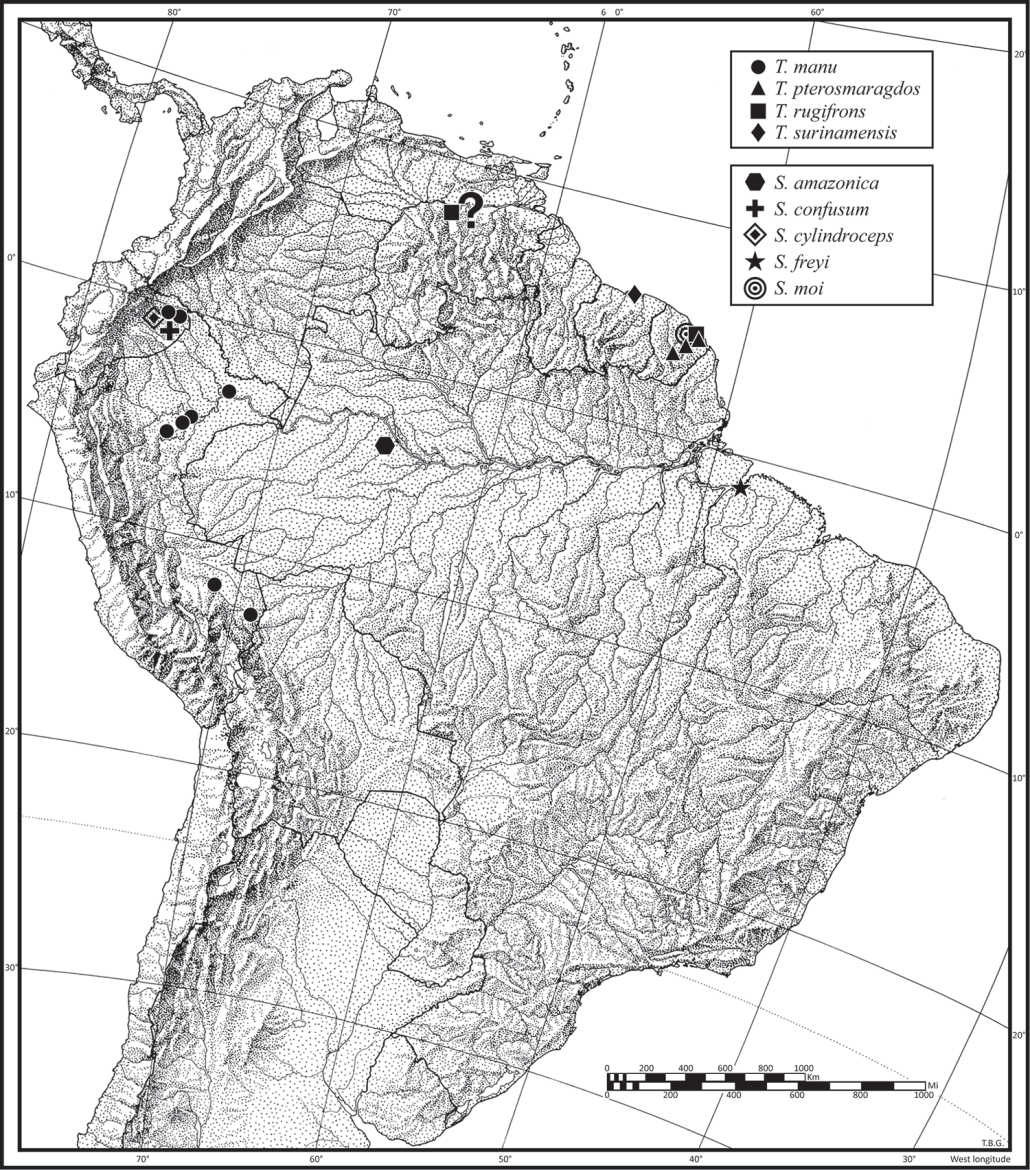
Distribution map for known localities of *Thoasia* and *Straneotia* species.

Slim arboreal carabid beetles Figs 4 , 5 , 6 , 7

Straneotia Mateu, 1961:165.

######## Type species.

*Straneotia freyi* Mateu, 1961, by original designation. Type locality. **Brazil**, Pará, Belém. The specimen is not in the Naturhistorisches Museum in Basel (G. Frey collection) as stated by Mateu, nor is it in the Muséum national d’Histoire naturelle, Paris (MHNP) where Mateu’s collection was deposited after his death.

######## Diagnosis.

(cf. Figs 4–6). Neck markedly broad, as wide as prothorax across apex of pronotum between lateral angles; eyes either somewhat flat, barely protruding beyond head outline, or moderately produced; frons flat, subtly rugose. Prothorax markedly narrow, barely wider than head; sides of pronotum narrowly reflexed throughout, cylindrical, hind angles subtly flared. Elytron at basal third very slightly transversely depressed, surface even, with slightly convex interval and striato-micropunctate interneurs. Flight wings clear. Basitarsus of mid and hind legs markedly elongate, coequal to length of tarsomeres 2–5. Male with two rows of adhesive setae on venter of tarsomeres 1–3; endophallus with flagellum; flagellum often extruded at apex; male with one pair of setae on sternum VI, females with two pairs.

######## Dispersal potential.

The wings are fully developed in adults of all known species, thus it is likely these beetles are moderate to strong flyers.

######## Geographic distribution.

A rare Neotropical genus known from Brazil, Ecuador, and French Guiana.

######## Ways of life.

Little is known about the species in this genus and that which is stated here is new. The newly collected specimens reported here were fogged from the canopy of rainforest trees.

######## Included species.

The species list below, as well as the arrangement of descriptions that follows is ordered alphabetically within two species groups.

######### *amazonica* species group

*S. amazonica* Mateu 1961, Brazil,

*S. confundis* Aldebron & Erwin, **sp. n.**, Ecuador

######### *freyi* species group

*S. cylindroceps* Erwin & Aldebron, **sp. n.**, French Guiana

*S. freyi* Mateu, 1961, Brazil

*S. moi* Aldebron & Erwin, **sp. n.**, Ecuador

######### Key to the species of *Straneotia* Mateu, 1961

**Table:** 

1	Adults with head normal with hemispherical eyes and short angulate gena to a broad neck	**2**
1’	Adults with head elongate and eyes somewhat flattened	**3**
2(1)	Adults with pronotal base at least as broad as widest point	***S. confundis* , sp. n.**
2’	Adults with pronotal base narrowing behind anterior pair of lateral setae	***S. amazonica* Mateu**
3(1’)	Head behind eye about 3 times length of eye	**4**
3’	Head behind eye about 1.2 times length of eye	***S. cylindroceps* , sp. n.**
4(3)	Adults with pronotal base much wider than apex, pronotum not appearing tubular; ligula with setal pores separated by at least the width of their diameter	***S. freyi* Mateu**
4’	Adults with pronotal base about subequal in width with that of apex, pronotum appearing tubular; ligula with setal pores close-spaced	***S. moi* , sp. n.**

###### *amazonica* species group

The most distinctive attribute of the only currently known species in this group is that the head is normal and somewhat depressed and the narrow pronotum is not tubular. Adults are mostly pale in coloration with dark areas in the basal third of the elytron. Male phallus apex moderately elongate, narrowed, spatulate (in *S. confundis*, *S. amazonica* male unknown).

####### Straneotia amazonica

Taxon classificationAnimaliaColeopteraCarabidae

Mateu, 1961

Amazon slim arboreal carabid Figs 5D , 7

Straneotia amazonica Mateu, 1961:166.

######## Holotype.

(Female): Type locality. **Brazil**, Amazonas, Tefé (Ega) (MHNP).

Azadeh Taghavian at the Muséum national d’Histoire naturelle in Paris was unable to locate the specimen amongst the Mateu collection. Therefore, we believe the type has been lost and a neotype will be needed when additional specimens are found.

######## Derivation of specific epithet.

The epithet, *amazonica*, is a singular Latin feminine noun in apposition referring to the macrohabitat in which the holotype was found.

######## Proposed English Vernacular Name.

Amazon slim arboreal carabid.

######## Diagnosis.

With the attributes of the genus and *amazonica* species group as described above and adults without a flared post-lateral margin of the pronotum.

######## Description.

(Fig. 5D). ***Habitus***: (Fig. 5D). *Size*: Length (ABL) small for genus, ABL = 5.5 mm.

Original description by Mateu (1961).

“Long. 5,5 mm. Alado. Misma coloración que la especie anterior, exceptuando que el color amarillo invade una parte más considerable de la superficie elitral y algo de la base y margenes laterales del pronoto. Las manchas oscuras de los cinco intervalos internos son más numerosas y visibles. Cabeza alargada, apenas convexa y deprimida entre los ojos. Estos son grandes y prominentes. Mejillas tan largas como los ojos, al principio relativamente poco estrechadas hasta cerca de la unión con el cuello, en donde se estrechan bastante bruscamente. Cuello moderadamente grueso pero formando una neta estrangulación en su unión con las mejillas. Surcos frontales paralelos. Los ojos estan bien separados entre si. La superficie de la cabeza es lisa, pero un tanto desigual a causa de las depresiones que se observan al lado de los ojos y por detrás del epístoma; éste es bastante alargado. Antenas finas llegando a sobrepasar ligeramente la base del pronoto. Diente labial redondeado en la extremidad. (Fig. 3b [in Mateu (1961)]). Pronoto alargado, cerca de vez y media más largo que ancho, convexo, bien que deprimido a lo largo del surco mediano. Borde anterior truncado, los ángulos anteriores redondeados. Los lados se ensanchan progresivamente en linea recta hasta la mitad de su longitud total desde donde se estrechan en linea oblicua hasta cerca de los ángulos posteriores que son obtusos. Base truncada. Reborde lateral ancho y reflejado hacia la base. Surco mediano fino. Elitros bastante convexos algo subcónicos y deprimidos sobre el disco a lo largo de la sutura. Húmeros ampliamente redondeados. Lados progresivamente estrechados hacia atrás. Apice sinuoso con el ángulo sutural algo prolongado. Estrías regulares, medianamente profundas y ligeramente punteadas. Intervalos subconvexos. Superficie elitral algo desigual. Reborde lateral estrecho, salvo a 5,2,1,6. Los poros intermedios casi equidistantes uno de otro y poco separados del grupo posterior. En realidad, sólo los cinco primeros constituyen una serie agregada. Metatibias rectas.”

######## Dispersal potential.

These beetles are macropterous and probably capable of flight.

######## Way of life.

The holotype was collected in the 1st trimester of 1878, the rainy season in the western Amazon Basin.

######## Other specimens examined.

None.

######## Geographic distribution.

(Fig. 7). This species is currently known from the western Amazon Basin.

######## Notes.

Because the type appears to be lost, we have provided Mateu’s original description and drawing.

####### Straneotia confundis

Taxon classificationAnimaliaColeopteraCarabidae

Aldebron & Erwin sp. n.

http://zoobank.org/D763A5D8-7F73-4409-9C48-4E69AEADA34C

Confusing slim arboreal carabid Figs 4C , 6B , 7

######## Holotype.

(Male): Type locality. **Ecuador**, Orellana, Yasuni National Park, –September 1998 (P. Araujo)(NMNH: ADP 152452).

######## Derivation of specific epithet.

The epithet, *confundis*, is a singular feminine adverb referring to the difficulty of our interpreting Mateu’s illustration of *S. amazonica* (see above).

######## Proposed English Vernacular Name.

Confusing slim arboreal carabid.

######## Diagnosis.

With the attributes of the genus and *amazonica* species group as described above and adults with a moderately narrow prothorax, the pronotum with markedly flared lateral margins in basal half and narrowly lateral explanation in apical half. Elytron with base of sutural interval and that of interval 3, 4, and 5 infuscated, and disc with a zig-zag infuscated mark on disc; disc base color testaceous, laterally (intervals 6-9) rufous.

######## Description.

(Figs 4C, 6B). ***Habitus***: (Fig. 4C). *Size*: See Appendix 1. Length (SBL) long for genus, ABL = 5.17 mm, SBL = 4.46 mm.

***Color***: *See* diagnosis above, and head and pronotal disc infuscated. ***Luster***: Very shiny. ***Microsculpture***: Mostly slightly stretched, shallowly impressed sculpticells, effaced from pronotum. ***Head***: Planar, perfectly smooth. Eye large, sub-hemispheric, and evenly rounded anteriorly, subtly more prolonged posteriorly. Antenna short, barely reaching humerus. Labrum subquadrate broadened slightly apically, truncate. Neck smooth. ***Prothorax***: Pronotum moderately narrow, disc centrally depressed with moderately dense and transverse rugae. Lateral margins moderately explanate and obtusely rounded medially then slightly arcuate to obtusely flared hind angle, base medially slightly produced and rounded. ***Pterothorax***: Normal for Agrina, fully winged. Elytron intervals flat, 3 and 5 with two discal unisetiferous punctures, side margin moderately explanate middle. Elytron broad and moderately long, moderately wider than the pronotum at the broadest part, apex arco-truncate, distal corner obtusely rounded with sutural corner narrowly rounded, disc more or less planar, basal third slightly depressed. All interneurs moderately impressed. ***Legs***: Femur and tibia normal, unmodified; basitarsus elongate, longer than tarsomeres 2-4 combined, fourth tarsomere markedly bilobed and with tarsal pad of setae. Claws pectinate. *Abdomen*: Glabrous with normal ambulatory setae on sterna 3-5; male with one pair of ambulatory setae on sternum VI located at extreme posterior corners. ***Male genitalia***: Phallus (Fig. 6B) with ostium of 1/6 its length, apex moderately short, narrowly pointed, tip rounded; endophallus with thick flagellum (obvious in illustration), flagellum not barbed. Parameres asymmetric, right very small, left larger. ***Female genitalia***: Unstudied, but likely similar to *S. cylindroceps* (cf. Fig. 6A).

######## Dispersal potential.

These beetles are macropterous and probably capable of flight.

######## Way of life.

The holotype was obtained with insecticidal fogging techniques from the canopy of terra firme lowland rainforest in September, the transition season between rainy and dry seasons in the area.

######## Other specimens examined.

None.

######## Geographic distribution.

(Fig. 7). This species is currently known from the Amazonian lowlands in the Yasuní area of northeastern Ecuador.

###### *freyi s*pecies group

The most distinctive attribute of species in this group is that the pronotum is somewhat tubular with narrow lateral explanations and the nearly flat eyes.

####### Straneotia freyi

Taxon classificationAnimaliaColeopteraCarabidae

Mateu, 1961

Frey’s slim arboreal carabid Figs 5A , 5B , 5C , 7

Straneotia freyi Mateu, 1961:165

######## Holotype.

(Female): Type locality. **Brazil**, Pará, Belém (G. Frey)(G. Frey Museum). Dr. Eva Sprecher-Uebersax informs us that the type is not in the Naturhistorisches Museum in Basel, Switzerland, the current home of the Frey collection. Likewise, Azadeh Taghavian at the Muséum national d’Histoire naturelle in Paris was unable to locate the specimen amongst the Mateu collection. Therefore, we believe the type has been lost and a neotype will be needed when additional specimens are found.

######## Derivation of specific epithet.

The epithet, *freyi*, is an eponym based on the family name of G. Frey who established the G. Frey Museum, Tutzing, Germany, and in which the holotype was originally deposited.

######## Proposed English Vernacular Name.

Frey’s slim arboreal carabid.

######## Diagnosis.

With the attributes of the genus and *freyi* species group as described above and adults with a markedly elongate head behind the eyes and five infuscated patches on otherwise unicolorous elytra.

######## Description.

(Figs 5A, 5B, 5C). ***Habitus***: (Fig. 5A). *Size*: See Appendix 1. Length (ABL = 7.5) long for genus.

Original description by Mateu (1961):

“Long. 7,5 mm. Alado. De un color rojizo y amarillento a lo largo de la sutura, por detrás del escudete, sobre los cinco primeros intervalos hasta sobrepasar la zona discal. Patas y antenas amarillas, mandíbulas rojizas. Palpos pardo-rojizo. Sobre los intervalos 1-3-4 se observan por delante del disco algunas manchitas irregulares de un color rojoparduzco oscuro. (fig. 3a in Mateu, 1961 [5B herein]). Cabeza muy larga, cilíndrica y paralela con los ojos grandes y completamente encajados en las sienes sin sobresalir del resto de la cabeza. Vistos dorsal mente los ojos esta n aproximados entre si dada la estrechez de la cabeza, la frente es asimismo muy estrecha. Sienes muy largas, casi el doble más largas que los ojos y practicamente paralelas y no estrechadas. Cuello grueso, ligeramente más grueso que las sienes en su unión con éstas y apenas estrechado hacia atrás. Surcos frontales paralelos. Epístoma alargado. Antenas finas, relativamente cortas, alcanzando sólo la base del pronoto. Superficie de la cabeza lisa, aparte de algunas arrugas débiles, cortas, transversales y paralelas entre sí. Diente labial subtruncado en la extremidad. Pronoto largo, estrecho, casi cilíndrico, por delante truncado y algo más ancho que el cuello; los lados se estrechan ligeramente al principio, ensanchándose algo poco después, hasta la inserción de la seda marginal situada por detrás de la zona discal; luego siguen ensanchándose hasta cerca de los ángulos posteriores en donde el pronoto se ensancha bruscamente alcanzando su mayor anchura en los mismos ángulos; éstos son muy obtusos. Base bisinuada. Surco mediano poco profundo. Superficie pronotal cubierta de arrugas transversales perpendiculares al surco mediano y equidistantes una de otra. Canal lateral estrecho, sólo algo reflejado por delante de los ángulos posteriores. Elitros convexos, algo subcónicos, anchos por delante y bien estrechados por detrás. Apice escotado, con el ángulo sutural prolongado. Estrías regulares, moderadamente profundas y punteadas. Intervalos planos. Superficie elitral algo desigual a causa de la depresión que existe entre las zonas escutelar y discal. Canal lateral estrecho en los húmeros, ensanchándose luego por detrás de éstos, para otra vez estrecharse hacia la mitad de la longitud elitral y desde este punto continuar estrechándose progresivamente hasta el ápice. Serie umbilicada de 16 poros setiformes distribuidos así: 6-1-1-8.”

***Male genitalia***: Unknown. ***Female genitalia***: Not investigated, likely similar to that of *S. cylindroceps* (cf. Fig. 6A).

######## Dispersal potential.

These beetles are macropterous and probably capable of flight.

######## Way of life.

Judging from the capture of adults of the following new species, this too is an arboreal species in rainforest canopies. The type was collected in December, the early rainy season in Belém.

######## Other specimens examined.

None.

######## Geographic distribution.

(Fig. 7). This species is currently known only from the type locality in the Amazonian lowlands near Belém, Brazil, at the confluence of the river.

######## Notes.

Because the type appears to be lost, we have provided Mateu’s original description and drawings.

####### Straneotia moi

Taxon classificationAnimaliaColeopteraCarabidae

Aldebron & Erwin sp. n.

http://zoobank.org/456D8B83-AABA-4A11-B656-1BE7A94A0736

Snake-head arboreal carabid Figs 4B , 7

######## Holotype.

(Female): Type locality. **French Guiana**, Cayenne, Commune de Roura, RN2, PK22, Montagne des Chevaux, 4.7127°N, 52.3966°W, 90m, 9 December 2012 (S. Brûlé, PH. Dalens, E. Poirier)(NMNH: ADP141236).

######## Derivation of specific epithet.

The epithet, *moi*, comes from the Wayampi word for snake, referring to the snake-like appearance of the species head. The Wayampi are indigenous to French Guiana (Payne 1999).

######## Proposed English Vernacular Name.

Snake-head arboreal carabid.

######## Diagnosis.

With the attributes of the genus and the *freyi* species group as described above and adults with testaceous, brown, and rufous hues on elytra, each elytron with irregular testaceous discal pattern, interrupted by narrow, transverse waves of dark brown, with basal third, rufous. Head behind eyes almost two times more than length of eye.

######## Description.

(Fig. 4B). ***Habitus***: (Fig. 4B). *Size*: See Appendix 1. Length (SBL) average for genus, ABL = 6.69 mm, SBL = 5.35 mm.

***Color***: *See* diagnosis above. ***Luster***: Elytra, forebody, and head markedly shiny. ***Microsculpture***: Elytron with mostly isodiametric and slightly stretched and moderately impressed sculpticells. Head and pronotum transversely microsculptured. ***Head***: Frons with two pairs of carinae extending from base of clypeus, converging to produce a V-shape in line with the beginning of the eye. Rugae of lateral occiput and neck mostly transverse, very finely etched. Eye not produced, barely convex. Antennae moderately long, reaching slightly behind humerus. Labrum large, spatula-shaped, with anterior angles broader than posterior angles. Neck finely transversely rugose at sides. Mouthparts distinct from *S. freyi* with labial palpomeres apically truncate and ligula markedly narrow with pair of setal pores medially close-spaced, with less than the width of their diameter between them. ***Prothorax***: Pronotum very narrow, cylindrical with slight lateral explanation; disc centrally narrowly planar, marked by a faint testaceous stripe and dense transverse rugae. Hind angles acute, subtly wider. ***Pterothorax***: Normal for Agrina, fully winged, flight wings translucent. Elytron intervals 3, 5, and 7 with numerous discal setae, intervals slightly convex, side margin moderately explanate. Elytron moderately broad and short, tapering markedly towards apex, markedly wider than the pronotum at the broadest part, apex truncate, markedly sinuate with distal corner obtusely rounded, disc not significantly convex, basal third slightly depressed. All interneurs well-impressed and micropunctate. ***Legs***: Normal for Agrina, no unique modifications. ***Abdomen***: Glabrous except normal ambulatory setae on sterna 3–5; female with two pairs of ambulatory setae on sternum VI. ***Male genitalia***: Unstudied. ***Female genitalia***: Unstudied but likely similar to that of *S. cylindroceps* (cf. Fig. 6a).

######## Dispersal potential.

These beetles are macropterous and probably capable of flight.

######## Way of life.

Adults, as supported by the collection of the type specimen by malaise trap, are most likely a canopy dwelling species of tropical rainforests. This species was collected in December, the beginning of a shorter, two month rainy season near the coast of French Guiana.

######## Other specimens examined.

No other specimens examined.

######## Geographic distribution.

(Fig. 7). French Guiana.

####### Straneotia cylindroceps

Taxon classificationAnimaliaColeopteraCarabidae

Erwin & Aldebron sp. n.

http://zoobank.org/4DBF42D9-8645-432E-A54F-9FFAF49D8125

Tube-headed slim arboreal carabid Figs 4A , 6A , 7

######## Holotype.

(Female): Type locality. **Ecuador**, Orellana, Reserva Etnica Huaorani, 39 km S Pompeya, Estación Científica Yasuní, Onkone Gare Camp, Erwin Piraña Plot: transect 7, station 8, 0.6556°S, 76.4475°W, 220–250m, 7 October 1995 (TL Erwin, et al.)(NMNH: ADP100396).

######## Derivation of specific epithet.

The epithet, *cylindroceps*, is a Latinized singular feminine adjective meaning “cylindrical head” referring to the shape of the elongate head behind the eyes.

######## Proposed English Vernacular Name.

Tube-headed slim arboreal carabid.

######## Diagnosis.

With the attributes of the genus and *freyi* species group as described above and adults with subtle violaceous tint on elytra, each elytron with three slightly elongate discal spots and interval two contrastingly pale in basal half. Head behind eyes about 1.2 times more than length of eye.

######## Description.

(Figs 4A, 6A). ***Habitus***: (Fig. 4A). *Size*: See Appendix 1. Length (SBL) small for genus, ABL = 5.47–5.77 mm, SBL = 5.22–5.23 mm.

***Color***: *See* diagnosis above. ***Luster***: Elytra with metallic violaceous highlights; forebody and head markedly shiny. ***Microsculpture***: Elytron with mostly isodiametric and slightly stretched and moderately impressed sculpticells. Head and pronotum devoid of microsculpture. ***Head***: Rugae of lateral occiput and neck mostly transverse, very finely etched; frons rugae more angulate. Eye not produced, barely convex. Antenna moderately long, reaching slightly behind humerus. Labrum small, rectangulate, shallowly emarginate. Neck finely transversely rugose at sides. ***Prothorax***: Pronotum very narrow, cylindrical with slight lateral explanation; disc centrally narrowly planar with dense transverse rugae. Hind angles obtusely acute, base medially subtly produced. ***Pterothorax***: Normal for Agrina, fully winged, wings clear. Elytron intervals 3, 5, and 7 with numerous discal setae, intervals slightly convex, side margin moderately explanate. Elytron moderately broad and short, moderately wider than the pronotum at the broadest part, apex obliquely truncate, markedly sinuate with distal corner obtusely rounded, disc not significantly convex, basal third slightly depressed. All interneurs well-impressed and micropunctate. ***Legs***: Normal for Agrina, no unique modifications. ***Abdomen***: Glabrous except normal ambulatory setae on sterna 3–5; female with two pairs of ambulatory setae on sternum VI. ***Male genitalia***: Unknown. ***Female genitalia***: (Fig. 6A). Ovipositor with broad spatulate laterotergite (lt) and two gonocoxites (gc 1, gc 2); gonocoxite 1 robust and apicolaterally aetose; gonocoxite 2 apically tapered, base (b) medium-size coequal in width with blade (bl) which is moderately elongate, apically obliquely narrowed and with several short robust and fine apical ensiform setae (des), several robust subapical ensiform setae, without ventral preapical nematiform setae. Reproductive tract proximally with moderately long, broad bursa copulatrix (bc), continuous at its distal end with common oviduct and long robust corrugated spermatheca (sp) distal to villous canal; spermathecal gland unknown (missing in dissection); spermathecal gland duct (sgd) very slim, attached to oviduct at base of its broadened portion.

######## Dispersal potential.

These beetles are macropterous and probably capable of flight.

######## Way of life.

Judging from the capture of the adults of new species, it is an arboreal species in rainforest canopies. The type and paratype were collected in February, the dry season in the Yasuní area of northeastern Ecuador. Members of this species have been recorded from the intersecting canopies of the following tree species using insecticidal fogging techniques: *Lacistema nena* cf.; *Guatteria glaberrima* cf.; *Inga auristellae* cf.; *Iriartea deltoidea*; *Eschweilera coriacea* cf.; *Warscewiczia cordata* cf.; *Virola obovata*; Astrocaryum murumuru var. urostachys; *Protium* “grand” sp. n.

######## Other specimens examined.

**Ecuador**: Orellana, Reserva Etnica Huaorani, 39 km S Pompeya, Estación Científica Yasuní, Onkone Gare Camp, Erwin Piraña Plot: transect 8, station 3, 0.6551°S, 76.4403°W, 220–250m, 8 February 1996 (TL Erwin, et al.)(NMNH: ADP 138079, female paratype).

######## Geographic distribution.

(Fig. 7). This species is currently known only from the type locality in the Yasuní area of northeastern Ecuador.

######## Notes.

The holotype is held in trust at NMNH until the completion of our Cryptobatida Group studies and then will be deposited in Escuela Politécnica Nacional, Quito, Ecuador (MHNEPN).

## Summary and future directions

Most of the 144 specimens used in this study were taken from the rainforest canopy, or upper understory, using insecticidal fogging techniques, or in flight intercept traps (windowpane or malaise).

In regard to the genus *Hyboptera*, with adults having many similar physical attributes as those in *Thoasia* (Erwin and Henry 2017), recent discoveries of several new *Hyboptera* species in remote parts of the upper Amazon Basin suggests that further sampling in such areas will also increase the species richness of *Thoasia*.

As noted by Erwin and Henry (2017), “Adults of the (currently) monobasic *Thoasia* Liebke 1939 are exceedingly common in canopy fogging samples (Erwin 1991); however, nothing is known about their way of life and they are only known with precise location from foggings in Perú and Ecuador, and FIT samples in French Guiana. Feeding specializations such as those hypothesized herein for adult *Hyboptera* and *Hybopteroides* and commonality of morphological attributes offer a fertile field of study on *Thoasia* for coleopterists eager to spend long periods of time in the rainforest canopies.”

*Straneotia* adults are very rarely collected and, so far, only by fogging the canopy. It is highly likely that many more species are yet to be discovered in the Amazonian rainforests.

## Supplementary Material

XML Treatment for Thoasia

XML Treatment for Thoasia rugifrons

XML Treatment for Thoasia surinamensis

XML Treatment for Thoasia pterosmaragdos

XML Treatment for Thoasia manu

XML Treatment for Straneotia

XML Treatment for Straneotia amazonica

XML Treatment for Straneotia confundis

XML Treatment for Straneotia freyi

XML Treatment for Straneotia moi

XML Treatment for Straneotia cylindroceps

## Appendix 1

Morphological measurements and ratios for adults of species of *Thoasia* Liebke 1939. All values are in millimeters. Apparent body length (ABL) and standard body length (SBL) are also provided in the descriptions. Means provided for ratios are harmonic means, as we believe they better reflect the central tendency than the arithmetic mean.

### *rugifrons* species group

**A.** *Thoasia rugifrons* Liebke.

	Males (N = 1)		Females (N = 1)	
	Range	Measure	Range	Measure
Total Length (SBL)		4.163		4.109
Maximum Width (MW)		1.903		1.776
Width of Head (HW)/Width of Left Elytron (EW)		1.025		1.060
Pronotum: Width (at widest part) (PW)/ Length (PL)		1.154		1.161
Length of Pronotum (PL)/ Length of Head (HL)		1.174		1.243
ABL		4.550		4.300

**B.** *Thoasia surinamensis* Erwin & Aldebron, sp.n.

	**Males (N = 0)**		**Females (N = 1)**	
	**Range**	**Mean**	**Range**	**Measure**
Total Length (SBL)				4.116
Maximum Width				1.852
Width of Head/Width of Left Elytron				1.013
Pronotum: Width (at widest part)/ Length				1.076
Length of Pronotum/ Length of Head				1.236
ABL				4.72

**C.** *Thoasia pterosmaragdos* Aldebron & Erwin, sp.n.

	Males (N = 2)		Females (N = 1)	
	Range	Mean	Range	Measure
Total Length (SBL)	4.208–4.349	4.279		4.388
Maximum Width	2.02–2.018	2.019		2.110
Width of Head/Width of Left Elytron	0.977–0.996	0.987		0.922
Pronotum: Width (at widest part)/ Length	1.233–1.254	1.243		1.172
Length of Pronotum/ Length of Head	1.209–1.228	1.219		1.248
ABL	4.970–5.070	5.020		5.110

### *manu* species group

**A.** *Thoasia manu* Erwin & Aldebron, sp.n.

	Males (N = 14)		Females (N = 19)	
	Range	Mean	Range	Mean
Total Length (SBL)	3.911–4.513	4.218	4.068–4.747	4.441
Maximum Width	1.889–2.286	2.052	1.833–2.332	2.132
Width of Head/Width of Left Elytron	0.857–1.012	0.965	0.883–1.101	0.966
Pronotum: Width (at widest part)/ Length	1.149–1.404	1.243	1.151–1.399	1.247
Length of Pronotum/ Length of Head	1.224–1.388	1.275	1.161–1.464	1.288
ABL	4.420–5.010	4.715	4.640–5.220	4.930

## Appendix 2

Morphological measurements and ratios for adults of species of *Straneotia* Mateu 1961. All values are in millimeters. Apparent body length (ABL) and standard body length (SBL) are also provided in the descriptions. Means provided for ratios are harmonic means, as we believe they better reflect the central tendency than the arithmetic mean.

### *amazonica s*pecies group

A. *Straneotia amazonica* Mateu Note: no specimens available for measurement

**B.** *Straneotia confundis* Aldebron & Erwin, sp.n.

	Males (N = 0)		Females (N = 1)	
	Range	Mean	Range	Measure
Total Length (SBL)				4.462
Maximum Width				1.996
Width of Head/Width of Left Elytron				0.878
Pronotum: Width (at widest part)/ Length				0.993
Length of Pronotum/ Length of Head				1.417
ABL				5.17

### *freyi s*pecies group

A. *Straneotia freyi* Mateu Note: no specimens available for measurement

**B.** *Straneotia moi* Aldebron & Erwin, sp.n.

	Males (N = 0)		Females (N = 1)	
	Range	Mean	Range	Measure
Total Length (SBL)				5.352
Maximum Width				2.090
Width of Head/Width of Left Elytron				0.682
Pronotum: Width (at widest part)/ Length				0.650
Length of Pronotum/ Length of Head				1.664
ABL				6.69

**C.** *Straneotia cylindroceps* Erwin & Aldebron, sp.n.

	Males (N = 0)		Females (N = 2)	
	Range	Mean	Range	Mean
Total Length (SBL)			5.216–5.229	5.223
Maximum Width			2.296–2.514	2.405
Width of Head/Width of Left Elytron			0.626–0.699	0.662
Pronotum: Width (at widest part)/ Length			0.825–0.828	0.826
Length of Pronotum/ Length of Head			1.548–1.579	1.563
ABL			5.470–5.770	5.62
